# Evaluation of Infectious Disease Knowledge in Obstetrics and Gynecology and the Effects of Varying Durations of Training

**DOI:** 10.1155/S1064744993000274

**Published:** 1993

**Authors:** Mark G. Martens

**Affiliations:** Division of Infectious Diseases Department of Obstetrics and Gynecology University of Texas Medical Branch Galveston TX 77555-0587 USA

## Abstract

*Objective:* The amount, origin, and resources of infectious disease knowledge in the field ofobstetrics and gynecology were investigated. If this knowledge is lacking, the exact length of the specific infectious disease training during residency should be defined to meet the ever-increasing knowledge required in training.

*Methods:* A 50-question test was developed by one faculty member utilizing questions that incorporated the basic sciences of microbiology and pharmacology and clinical knowledge of infectious diseases in the area of obstetrics and gynecology. Multiple choice and matching questions were structured so as to ascertain the source of the knowledge, including medical school curriculum, recent journal articles, and clinical experience.

*Results:* The test was given yearly to all students and residents on the Obstetric and Gynecology Service over a period of 2 year's. The questions were the same for each group, but were reshuffled each exam period. Three hundred and seven tests were properly administered and recorded. There was no statistical improvement in any successive year’s scores unless specific infectious disease training occurred. Increasing improvement in scores was noted, with an increasing duration of infectious disease training specific for obstetrics and gynecology, beginning at 2 weeks (22% improvement), 4 weeks (30% improvement), and 6 weeks (31% improvement) (*P* = .05–.001). Basic science questions were most frequently answered correctly by medical students and early residents, while correctly answered clinical questions correlated with increasing clinical experience except in the area of ambulatory care.

*Conclusions:* The infectious disease knowledge of residents in obstetrics and gynecology can be improved with 4 weeks of intensive training. Re-exposure to basic science knowledge and improved training in ambulatory care in this resident group appear to be necessary. This test or similar tests can be helpful in defining areas of deficiencies and their possible remedies.


Infectious Diseases in Obstetrics and Gynecology 1:118–122 (1993)

© 1993 Wiley-Liss, Inc.

### Evaluation of Infectious Disease Knowledge in Obstetrics and Gynecology and the Effects of Varying Durations of Training

**Mark G. Martens**

*Division of Infectious Diseases, Department of Obstetrics and Gynecology, University of Texas Medical Branch, Galveston, TX*

Abstract

***Objective:*** The amount, origin, and resources of infectious disease knowledge in the field of obstetrics and gynecology were investigated. If this knowledge is lacking, the exact length of the specific infectious disease training during residency should be defined to meet the ever-increasing knowledge required in training.

***Methods:*** A 50-question test was developed by one faculty member utilizing questions that incorporated the basic sciences of microbiology and pharmacology and clinical knowledge of infectious diseases in the area of obstetrics and gynecology. Multiple choice and matching questions were structured so as to ascertain the source of the knowledge, including medical school curriculum, recent journal articles, and clinical experience.

***Results:*** The test was given yearly to all students and residents on the Obstetric and Gynecology Service over a period of 2 years. The questions were the same for each group, but were reshuffled each exam period. Three hundred and seven tests were properly administered and recorded. There was no statistical improvement in any successive year's scores unless specific infectious disease training occurred. Increasing improvement in scores was noted, with an increasing duration of infectious disease training specific for obstetrics and gynecology, beginning at 2 weeks (22% improvement), 4 weeks (30% improvement), and 6 weeks (31% improvement) ($P$ = .05–.001). Basic science questions were most frequently answered correctly by medical students and early residents, while correctly answered clinical questions correlated with increasing clinical experience except in the area of ambulatory care.

***Conclusions:*** The infectious disease knowledge of residents in obstetrics and gynecology can be improved with 4 weeks of intensive training. Re-exposure to basic science knowledge and improved training in ambulatory care in this resident group appear to be necessary. This test or similar tests can be helpful in defining areas of deficiencies and their possible remedies. © 1993 Wiley-Liss, Inc.

Key words

Ob-Gyn residency, infectious disease training, subspecialty evaluation

Infectious disease training is usually accomplished formally in medical school for persons entering the field of obstetrics and gynecology or the surgical specialties. Obstetrics and gynecology residents are expected to expand upon this knowledge base during their training through a variety of methods including faculty interaction, lectures, courses, conferences, and individual reading. The efficacy of each of these methods has not been assessed, nor have the specific areas of expertise or deficiency in residency been fully examined. If obstetrics and gynecology-specific infectious disease training is

Address correspondence/reprint requests to Dr. Mark G. Martens, Division of Infectious Diseases, Department of Obstetrics and Gynecology, University of Texas Medical Branch, Galveston, TX 77555-0587.

Received March 31, 1993
Accepted September 1, 1993

available, the proper duration and efficacy of such training are also not known. An understanding of the appropriate duration of subspecialty training is imperative, in light of the ever-increasing fund of knowledge and experience that a residency program must cover in 4 years. Redundant training is as much a waste of valuable time and effort as is ineffective training.

The fund of knowledge of infectious diseases in obstetrics and gynecology was measured in medical students, residents, and faculty. In addition, the areas of adequate or deficient specialty-specific infectious disease knowledge were determined in students and obstetrics and gynecology residents before and after varying durations of intensive training.

### MATERIALS AND METHODS

A 50-question test was developed by the author that incorporated questions related to the basic sciences of microbiology and pharmacology as well as the clinical knowledge of infectious diseases in the area of obstetrics and gynecology. The test questions were multiple choice or matching.

Twenty-one questions were related to the microbiology of pelvic infections. Ten questions were related to the pharmacology of infections and their treatment. The remaining 19 questions were based upon clinical situations that were deemed to be reflective of the current clinical practice on the university service.

The questions were also structured to ascertain the source of the infectious disease knowledge. Sixteen questions were derived from information that was believed to be acquired in medical school. Thirteen questions required correlation with clinical experience in order to determine the correct answer. The remaining 21 questions could only be answered correctly if the individual had read the recent literature (of the past 2 years) or had specifically attended lectures or received training in a subspecialty. A representative sample of 5 questions is given in Table 1.

The test was administered in a monitored setting at the University of Texas Medical Branch in Galveston to all 3rd-year medical students at the end of their obstetrics and gynecology rotation; to all 4th-year medical students before and after their 1-week rotation on the obstetrics and gynecology infectious disease service; to obstetrics and gynecology residents at all four levels of training annually for 2 years; and to university faculty. The tests were reviewed by physician members of the Infectious Diseases Society for Obstetrics and Gynecology (IDSOG). After validation, the remaining questions and answers were utilized, and the participating IDSOG group of physicians served as the control group. Half of the 3rd-year residents each year over the period of 2 years completed an obstetrics and gynecology infectious disease elective. The elective was comprised of 4 weeks of intensive experience including twice daily rounding for a minimum of 2 hours a day, reading requirements, consultations, and 2 hours of informal lectures a week. One-half of the 1990 2nd-year residents undertook a 6-week rounds-only infectious disease elective, while half of the current 3rd-year residents completed 2 weeks of infectious disease rounds. Repeat tests were given each year with the order of the questions being reshuffled each testing period.

**TABLE 1. Sample questions**

1. #2 The second most common organism found in cultures from women with urinary tract infection (UTI) is:
   a) *E. coli*
   b) Enterococci
   c) ***Staphylococcus saprophyticus***
   d) *Gardnerella vaginalis*
2. #5 Cefazolin's trade names include all but:
   a) Ancef
   b) **Ceftin**
   c) Kefzol
3. #11 Antibiotics that cross the placenta adequately include all but:
   a) Mezlocillin
   b) **Erythromycin**
   c) Cefizoxime
   d) Acyclovir (Zovirax)
4. #21 Each of these antibiotics has activity versus chlamydia except:
   a) Tetracycline
   b) Erythromycin
   c) **Cefoxitin**
   d) Clindamycin
5. #40 A patient comes to your office after noticing a nontender ulcer on her labia majora. Upon examination, you note a 2 × 2 shallow nontender ulcer, along with ipsilateral nontender enlarged inguinal lymph nodes. The next course of action is:
   a) Obtain RPR and treat per results
   b) **Arrange for darkfield examination**
   c) Start tetracycline, 500 mg PO QID
   d) Observe the patient for 1 week

**TABLE 2. Obstetrics and gynecology infectious disease test scores**

| Group | Initial test | | Repeat test | | P value |
|---|---|---|---|---|---|
| | No. | % Correct | No. | % Correct | |
| Medical students | | | | | |
| 3rd year | 91 | 44 | —[a] | — | — |
| 4th year | 72 | 42 | 60 | 53 | <.005 |
| Ob/Gyn residents | | | | | |
| 1st year | 9 | 58 | 7 | 62 | NS |
| 2nd year | 5 | 54 | 4 | 66[b] | .05 |
| | | | 4 | 70[c] | <.001 |
| 3rd year | 7 | 62 | 7 | 83[d] | <.001 |
| 4th year | 9 | 58 | — | — | — |
| University Ob/Gyn faculty | 9 | 56 | — | — | — |
| Pharmaceutical representatives | 8 | 52 | — | — | — |
| IDSOG[e] | 15 | 72 | — | — | |
| Total | 225 | | 82 | | |

[a]Tests not repeated.
[b]Following 2 weeks of infectious disease service rounding twice daily.
[c]Following 4 weeks of full-time infectious disease service.
[d]Following 6 weeks of full-time infectious disease service rounding only.
[e]Infectious Diseases Society for Obstetrics and Gynecology.

Test scores were analyzed using the paired Student's t-test, with a $P$ value $\leq 0.05$ achieving statistical significance.

### RESULTS

Three hundred seven tests were administered. Table 2 demonstrates the year-by-year results. Ninety-one tests were given to junior medical students following their 6-week rotation on obstetrics and gynecology. No specific infectious disease training was provided for these students except for one lecture on sexually transmitted diseases and one lecture on pelvic inflammatory disease. Their mean score at the end of their rotation was 44%. Fourth-year medical students took the test during their 4-week obstetrics and gynecology subspecialty rotation before and after their 1-week infectious disease rotation. Seventy-two students took the test prior to the rotation and obtained a mean score of 42%, demonstrating no statistical difference between the 3rd and 4th year of medical school. After the infectious disease rotation, 60 4th-year students had a mean score of 52% ($P < .001$).

Tests were given to all obstetrics and gynecologic residents 6 months into the 1990 academic year. Nine 1st-year obstetrics and gynecology residents scored a mean of 58%, five 2nd-year residents scored a mean of 53%, seven 3rd-year residents scored a mean of 62% and nine 4th-year residents scored a mean of 58%. An infectious disease service in obstetrics and gynecology was begun in 1990. Therefore, all residents were exposed to unlimited consultations of the infectious disease service and to a series of eight formal and two to six informal noon lectures over the period of a year. Interns, with a mean score of 58%, did not significantly improve their scores after 1 year of passive training ($P = .22$). Second-year residents underwent one of two regimens of formal infectious disease training. The first group had 2 weeks of infectious disease service rounding only, while the second group had 4 weeks of full-time service duty. Each group's score improved significantly from a mean of 54% before training ($P < .001$, respectively). However, there was no significant difference between the mean scores of 66% and 70% following the 2- or 4-week training period, respectively.

Seven 4th-year residents completed 6 weeks of infectious disease service rounds and significantly improved their test scores from 62% in 1990 to 82% in 1991 ($P < .001$). University obstetrics and gynecology faculty took the test and scored a mean of 56%; eight hospital pharmaceutical representatives took the test with a mean score of 52%, which was not statistically different from those of the initial tests from the resident group. Fifteen

IDSOG members scored a mean of 72%, and these scores served as the control.

Analysis of the areas of knowledge of the junior medical students demonstrated that the most common questions incorrectly answered dealt with the microbiology or antibiotic treatment that was specific to obstetrics and gynecology, with fewer than 5% of these questions answered correctly. The four questions most frequently answered correctly were related to basic microbiology and pharmacology, with greater than 90% of the students answering these questions accurately. Senior medical students' correct and incorrect answers followed similar patterns. The residents as a group were more knowledgable in the clinical areas, with greater than 70% of these questions answered correctly. However, answers in the areas of basic microbiology and pharmacology were correct less than 30% of the time. Surprisingly, fewer than 50% of questions pertaining to outpatient or office management of infections were answered correctly.

Answers requiring knowledge primarily obtained from reading the recent literature reflected less than 40% accuracy. IDSOG members had questions marked incorrect when subspecialty or regional differences or personal ongoing unpublished research affected their answers. Overall, IDSOG members initially scored statistically higher than all other groups, as expected ($P < .001$).

### DISCUSSION

Infectious disease training in medical school and in obstetrics and gynecology residency programs is often assumed to be acquired by exposure to the various aspects of individual specialties (such as maternal-fetal medicine or operative gynecology) and to the literature. Due to the diverse nature of obstetrics and gynecology, an integration of the basic sciences, especially microbiology and pharmacology, with the variety of acute, operative, and outpatient clinical situations must be accomplished. This integration of basic and clinical sciences traditionally occurs separately in medical school and residency. An ideal situation would be to combine both entities or at least reinforce them during the same training period.

Through the examination of the specific areas of expertise and deficiencies, it appears that much basic science knowledge is lost, as the average obstetrics and gynecology clerkship lasts only 7 weeks.[1] It is, however, replaced over the 4 years of residency by knowledge obtained from clinical experience. In this investigation, medical students from the 3rd to 4th year and obstetrics and gynecology residents from the 1st to the 4th year were not shown to acquire obstetrics and gynecology-specific infectious disease knowledge passively. Scores on the infectious disease test did not significantly improve for any year except when formal training occurred. Scores increased proportionally with longer durations of infectious disease training.

From the change in which questions were scored correctly over time, it is evident that re-exposure to basic science education would be helpful in expanding the fund of infectious disease knowledge for obstetrics and gynecology residents. This need appears early in the residency period and continues after residency.

Despite the improvement in scores with training of infectious disease faculty members, specific trends in knowledge lost and gained were identifiable from the test questions. Therefore, with direct attention, any institution should be able to correct these deficiencies. The unimpressive scores of the university faculty can be seen in the light of individual scores versus the entire faculty complementing each other in the training of the residency group. If specific departmental deficiencies are suspected, a method of evaluation through quality assurance records as proposed by Silberman may be utilized.[2] Maternal-fetal medicine faculty members were knowledgable in the infections of their subspecialty, whereas they performed less efficiently in gynecology areas. The converse was true for the gynecologist. Therefore, a concerted effort by the entire faculty should provide a complete spectrum of infectious disease knowledge for the resident in training. This should be accomplished with greater ease in the future as the number of medical school faculty continues to grow.[3] However, specific areas such as outpatient medicine need to be included. Re-education in the basic sciences, specifically microbiology and pharmacology, have already been identified as necessary, but will have to be specifically addressed by an alteration in the teaching curricula, as upper level residents and university faculty apparently have similar deficiencies. While these deficits are serious, fortunately they do not often result in poor patient care in a university setting, as there are usually several "layers" of care with supervision of each training level from student to resident to faculty. In addition graduated residents usually have partner-to-partner or other colleague contact to help in areas in which they are uncertain. Lastly, even if support systems fail, the isolated practicing physician is best trained in clinical matters, and weaknesses in basic science knowledge should not present a critical deficit. However, improving the education of medical students and residents is the important issue and concern.

In summary, infectious disease training in obstetrics and gynecology does not appear to expand with passive learning, but is replaced with clinical knowledge over the years of training from medical school through residency. Four to 6 weeks of exposure to a concentrated obstetrics and gynecology-specific infectious disease service appears to be the most effective method of training and achieves a knowledge base equivalent to that of recognized infectious disease experts. Alternative sources of training and education should directly address the basic sciences and outpatient medicine. However, each institution will have its own strengths and weaknesses that can be examined through testing, following which the deficiencies can be directly addressed.

### REFERENCES

1. Herbert WNP, Cummings RV, Droegemueller W: Profile of student clerkship administration in obstetrics and gynecology. Obstet Gynecol 76:153–155, 1990.
2. Silberman L: Quality assurance in obstetrics: A model. Obstet Gynecol 76:466–470, 1990.
3. Pearse WH, Graham KK: Trends in obstetric-gynecologic academic manpower and research. Obstet Gynecol 78:141–143, 1991.
